# Food insecurity, diabetes, and perceived diabetes self-management among Latinos in California: Differences by nativity and duration of residence

**DOI:** 10.1016/j.pmedr.2022.101856

**Published:** 2022-06-09

**Authors:** Brandon Osborn, Sandra S. Albrecht, Nancy L. Fleischer, Annie Ro

**Affiliations:** a Program in Public Health, University of California, Irvine, Anteater Instruction and Research Building (AIRB), Room 2030, 653 E. Peltason Road, Irvine, CA 92697-3957, United States; b Department of Epidemiology, Columbia University Mailman School of Public Health, 722 West 168th Street, Room 703, New York, NY 10032, United States; c Center for Social Epidemiology and Population Health, Department of Epidemiology, School of Public Health, University of Michigan, 1415 Washington Heights, Ann Arbor, MI 48109-2029, United States

**Keywords:** Food insecurity, Diabetes, Latinos, Nativity, Duration

## Abstract

We examined associations between food security (FS) status and type 2 diabetes (T2D) prevalence and perceived T2D self-management by nativity and US duration of residence among Latinos living in California. We used the California Health Interview Survey (2012–2017) and included Latinos who lived below 200% of the federal poverty line (n = 16,254) and for our management outcome, those with T2D (n = 2284). Latinos with low FS (OR = 1.44, 95% CI 1.14–1.83) or very low FS (OR = 1.87, 95% CI 1.33–2.61) had a higher odds of T2D compared to their food-secure counterparts. When stratified by *nativity/duration* in the US, US-born Latinos and Latino immigrants with >10 years duration had a higher odds of T2D if they reported low FS (US-born: OR = 1.60, 95% CI 1.02–2.52; >10 yrs: OR = 1.48, 95% CI 1.12–1.97) or very low FS (US-born: OR = 2.37, 95% CI 1.45–3.86; >10 yrs: OR = 1.78, 95% CI 1.15–2.76) compared to their food-secure counterparts. There was no association among immigrants with <10 years duration. For perceived T2D self-management, those with low or very low FS had lower odds of reporting proper management (OR = 0.56, 95% CI 0.36–0.86; OR = 0.46, 95% CI 0.26–0.83) compared to their food-secure counterparts. When stratified by *nativity*, the US-born did not differ in their perceived self-management by FS status, while immigrants with low or very FS had lower odds of perceived self-management (OR = 0.54, 95% CI 0.34–0.86; OR = 0.36, 95% CI 0.17–0.74), compared to their food-secure counterparts. Food insecurity may be an important contributor to T2D prevalence and perceived T2D self-management for Latino immigrants.

## 1 Introduction

The prevalence of diabetes in the United States (US) has continued to increase over the last 50 years; in 2019, an estimated 28.5 million adults—or 8.7% of the U.S. population – had diagnosed diabetes (Centers for Disease Control and Prevention, 2020). Type 2 diabetes (T2D) accounts for about 95% of these diabetes cases (Centers for Disease Control and Prevention, 2017). Latinos are disproportionally affected by T2D, with a higher prevalence of diagnosed diabetes (12.5%) compared to non-Hispanic whites (7.5%) during 2017–2018 (Centers for Disease Control and Prevention, 2020). This disparity continues to grow as people of Hispanic origin also have had a higher incidence of diagnosed diabetes (9.0 per 1,000 persons) compared to Non-Hispanic whites (5.4 per 1,000 persons) during 2017–2018 (Centers for Disease Control and Prevention, 2020).

Many social and environmental factors are associated with T2D. Among these, *food insecurity*, which is defined as a lack of access by all people at all times to enough food for an active, healthy life, has been found to be an important risk factor (Hill et al., 2013, USDA, 2017). Previous studies have found that individuals living in food-insecure households have a higher proportion of T2D when compared to their food-secure counterparts (Gucciardi et al., 2014, Essien et al., 2016, Seligman et al., 2007, Fitzgerald et al., 2011, Bawadi et al., 2012, Seligman et al., 2010). One study among low-income Americans found that participants with food insecurity were more than two times more likely to have T2D compared to their food-secure counterparts after adjusting for sociodemographic factors, physical activity level, and body mass index (Seligman et al., 2007). Food insecurity appears to be a particularly important T2D risk factor for Latinos. Not only is the prevalence of food insecurity considerably higher among Latino households (18.0%) than the national average (11.8%), but the link between food insecurity and T2D is stronger compared to other racial/ethnic groups (USDA, 2017). One study found that Latinas with very low food security were 3.3 more likely to have T2D compared to their food-secure counterparts even after controlling for employment, waist circumference, acculturation, and lifestyle characteristics (Fitzgerald et al., 2011, Seligman et al., 2007).

Food insecurity is also associated with poor glycemic control among people with T2D (Seligman et al., 2012, Berkowitz et al., 2013). The costs of glucose monitoring strips and prescription medication compete with costs for basic needs such as food and housing. Competing demands have been reported as a barrier to adherence to treatment plans among patients with T2D, regardless of race/ethnicity (Berkowitz et al., 2015). One study among chronically-ill adults found that medication underuse due to cost was significantly higher among food insecure individuals compared to food-secure individuals (Berkowitz et al., 2014). Similarly, a study among adults with T2D found that food-insecure participants had almost six times higher odds of scrimping their medications compared to their food-secure counterparts (Knight et al., 2016). Food-insecure individuals are less likely to fill their prescription, (Billimek and Sorkin, 2012) use new needles, and monitor their glucose levels regularly (Marjerrison et al., 2011, Seligman et al., 2011, Chan et al., 2012) since these compete with buying healthy foods and paying rent (Seligman et al., 2010, Seligman et al., 2010, Beryl Pilkington et al., 2010). Lastly, food-insecure individuals have reported difficulty affording a diabetic diet, have a lower diabetes-specific self-efficacy, and have higher emotional distress related to T2D (Seligman et al., 2012). Among people with T2D, Latinos also seem to have poorer control of their condition compared to other racial/ethnic groups, as measured by HbA1c value (Kaplan et al., 2013, Heisler et al., 2007).

Latinos’ disproportionate exposure to food insecurity puts them at a greater risk for T2D and poor management outcomes. Yet this association may not be uniformly experienced within the larger Latino population. Food insecurity is more prevalent among Latinos immigrants (24.4%) than their US-born counterparts (18%) (Food Insecurity and Diversity, 2017). T2D prevalence is further patterned by the length of time in the US (Schneiderman et al., 2014). The prevalence of T2D is highest among immigrants who have lived in the US for >15 years (18.8 percent) compared to recent immigrants (12.2 percent) and Latinos born in the US (14.5 percent) (Schneiderman et al., 2014). Although Latino immigrants have the lowest levels of T2D, they also have the highest levels of food insecurity (Food Insecurity and Diversity, 2017). Similar patterns have been found for obesity, with recent immigrants having the highest levels of food insecurity, but not displaying a significant association between food insecurity and obesity (Ryan-Ibarra et al., 2017). In contrast, food insecurity is associated with a higher likelihood of obesity among those born in the US and immigrants with longer duration in the US (Ryan-Ibarra et al., 2017). Although the prevalence of food insecurity and T2D each differ by nativity and duration in the US, this study examines whether the relationship between food insecurity and T2D differs by these factors among Latinos as well.

Currently, there are no studies examining the associations between food insecurity and T2D prevalence or glycemic control among Latinos by nativity or duration of stay in the US.

The countervailing trends of T2D prevalence and food insecurity among Latinos by nativity and duration underscore the importance of examining nativity and duration when examining the relationship between food insecurity, T2D, and perceived T2D self-management. In this paper, we examine the extent to which the relationship between food insecurity and T2D, as well as perceived T2D self-management, differs by nativity and duration of residence among Latinos living in California. We focus on California for several reasons. First, California has the highest foreign-born population of any state in the United States (Budiman, 2020). In 2018, 10.6 million immigrants lived in California, which makes up 24% of all immigrants in the United States (Budiman, 2020). This large population ensures a large enough sample to identify differences with the Latino population by nativity and duration. Second, among immigrant Latinos in California, estimates of food insecurity prevalence have ranged from 25.1% to 40.5% (Walsemann et al., 2017). These high prevalence rates contrast to nationwide estimates, where 24.4% of Latino immigrants have been found to be food insecure (Food Insecurity and Diversity, 2017).

## 2 Methods

We analyzed data from the 2012–2017 California Health Interview Survey (CHIS) (California Health Interview Survey | UCLA Center for Health Policy Research, 2017). The CHIS is a cross-sectional, random digit dial telephone survey, representative of California’s noninstitutionalized population, and is the largest state health survey in the nation. The CHIS is administered in multiple languages and oversamples minority racial/ethnic groups to ensure adequate numbers of participants from a variety of racial/ethnic backgrounds participate in the survey. We combined six waves of data to ensure a sufficient sample of Latinos born in and outside of the United States (total Latinos: n = 27,988). We chose to utilize the CHIS since California has the highest proportion of immigrants compared to other states in the United States (Budiman, 2020). We further restricted our sample to those who had a household income <200% of the federal poverty line, as this was the income threshold for the CHIS food insecurity questionnaire (n = 16,254). In the analyses in which we examined perceived T2D self-management, our sample was further restricted to those with T2D (n = 2,284). This study was determined to be non-human subjects research by the University of California, Irvine, Institutional Review Board.

## 3 Measures

### 3.1 Food security status

Food security status was measured using the validated United States Department of Agriculture Household Food Security Survey Module six-item form (Bickel et al., 2012). This form assesses self-reported food insecurity over the past 12 months. Raw scores ranging from 0 to 6 were generated by the affirmative responses to the questions. Food security status was classified according to the US Department of Agriculture’s (USDA) guidelines: 0–1, high or marginal food security; 2–4, low food security; 5–6, very low food security.

### 3.2 Diabetes

T2D was measured by two questions, “Has a doctor ever told you that you have diabetes?” and “type 1 or type 2 diabetes?”. T2D was distinguished if respondents answered “yes” and specified type 2 in their responses. Less than four percent (n = 148) of Latinos reported having been diagnosed with diabetes, but not specifying which type (type 1 or type 2). These cases were classified as missing as we could not determine whether they had type 1 or type 2 diabetes. The percent missing (4%) is below the range that is considered problematic (10% or more) for missing data biases (Schafer and Graham, 2002).

### 3.3 Perceived diabetes self-management

Perceived T2D self-management was measured by the question: “How confident are you that you can control and manage your diabetes?” This was only asked of respondents who had been told by a physician they had diabetes. Response items included very confident, somewhat confident, not too confident/not at all confident. For the purpose of our analyses, we dichotomized the categories to “very confident” versus all others to minimize social desirability bias.

### 3.4 Nativity/US duration

The CHIS uses a categorical variable to measure the duration of time living in the United States (US): <5 years, 5–9 years, 10–14 years, 15+ years. In addition, The CHIS measures nativity by asking respondents if they were born in the US: yes/no. We combined both of these measures into one categorical variable to measure nativity and duration of time living in the US: Born in the US (reference), Foreign-born with <10 years US duration, and Foreign-born with 10+ years US duration. There were no missing values for these variables. We used the nativity/duration combined variable when examining T2D prevalence. However, for the perceived T2D self-management outcome, we used the nativity variable of born within or outside the US due to the small sample size of Foreign-born Latinos with <10 years of duration of living in the United States with T2D.

### 3.5 Covariates

In multivariable analyses, we controlled for age in years; education (less than high school grad, high school grad, some college/college grad); income (<20,000, 20,000–29,000, and 30,000+); gender (female or male); health insurance (does not have health insurance, has health insurance); family type (single no children, married no children, married with children, single with children); and survey year to account for potential period effects and temporal trends of food insecurity (Walsemann et al., 2017). There was no variation in the questions and response options across the waves of the survey.

### 3.6 Statistical analysis

We calculated descriptive statistics stratified by nativity/US duration. We then conducted a series of logistic regressions. We first established the unadjusted association between food security status and the T2D outcome (prevalence or self-management), then controlled for all covariates. Similar to Ryan-Ibarra et al. (Schneiderman et al., 2014), we stratified our models examining T2D prevalence by nativity/US duration (Ryan-Ibarra et al., 2017). For our models examining perceived T2D self-management, we stratified by nativity only. For both sets of models, we stratified to assess whether the association between food insecurity and T2D and perceived T2D self-management is different by nativity/duration.

We performed all statistical analyses using STATA/IC 14 (StataCorp LP, College Station, TX, USA). We incorporated replicate weights using jackknife replications to account for the complex sampling design and appended and harmonized across the replicate weights to accommodate pooling across six survey waves. This led to population estimates that give equal weight to each year of data (UCLA Center for Health Policy Research, 2020). We assessed all models for multicollinearity; all Variance Inflation Factors remained below 10.

## 4 Results

### 4.1 Sample characteristics

A higher percentage of Latinos living in the US for 10 years or more reported having T2D (13.7%) compared to Latinos born in the US (8.2%) and those living here in the US for <10 years (3.3%) (Table 1). Latinos born in the US had the highest levels of high/marginal food security (62.6%), while immigrant Latinos had lower levels, though estimates did not differ by US duration (51.9% among ≥10 years, 51.6% among <10 years). However, the percentage of Latinos reporting very low food security was similar across all nativity/duration categories. Latinos born in the US had higher educational attainment and income compared to foreign-born Latinos. Among Latinos with T2D, a higher percentage of foreign-born Latinos reported having better perceived T2D self-management (56.2 % among ≥10 years, 52.4% among <10 years) than US-born Latinos (45.8), despite also having lower rates of health insurance.

**Table 1:** Weighted Sample Description, California Health Interview Survey 2012–2017 Latino Adults That Live Under 200% of the US Federal Poverty Line, N = 16,254.

	**Native Born (n = 5,475)**	**[95% CI]**		**Living in US < 10 Years (n = 1,173)**	**[95% CI]**		**Living in US ≥ 10 Years (n = 9,606)**	**[95% CI]**	
**Demographics**									
Mean Age (SE)	31.3(0.41)			31.6(0.53)			44.9(0.23)		
Gender, %									
Female	55.2	0.53	0.58	57.1	0.52	0.62	53.5	0.52	0.55
Male	44.8	0.42	0.47	42.9	0.38	0.48	46.5	0.45	0.48
Education, %									
Less than High School	17.3	0.15	0.20	56.7	0.52	0.61	66.1	0.64	0.68
High School Diploma	72.3	0.70	0.75	33.6	0.29	0.38	29.6	0.27	0.31
Some College+	10.4	0.09	0.12	9.7	0.07	0.13	4.4	0.04	0.05
Household total Annual Income, %									
$0–19,999	34.6	0.32	0.37	47.9	0.42	0.54	36.2	0.34	0.38
$20,000–29,000	25.0	0.23	0.27	27.2	0.23	0.32	28.7	0.26	0.31
$30,000+	40.4	0.38	0.43	24.9	0.19	0.32	35.2	0.33	0.37
Family Type, %									
Single No Kids	61.3	0.59	0.64	38.7	0.34	0.44	27.5	0.26	0.29
Married No Kids	7.4	0.06	0.09	7.9	0.06	0.11	19.8	0.19	0.22
Married with Kids	18.4	0.16	0.21	40.1	0.35	0.46	41.4	0.40	0.43
Single with Kids	12.8	0.11	0.15	13.3	0.10	0.17	11.2	0.10	0.13
Currently Has Health Insurance, %	80.9	0.79	0.83	54.2	0.49	0.60	70.5	0.68	0.72
**Food Security Status, %**									
High/Marginal Food Security	62.6	0.60	0.65	51.6	0.46	0.57	51.9	0.49	0.54
Low Food Security	23.8	0.22	0.26	35.4	0.30	0.41	34.8	0.33	0.37
Very Low Food Security	13.6	0.11	0.15	13.1	0.10	0.17	13.3	0.12	0.15
**Diabetes and Management, %**									
Type 2 Diabetes Prevalence,	8.2	0.07	0.10	3.3	0.02	0.06	13.7	0.12	0.15
Prevalence of Managing Diabetes Well*	45.8	0.37	0.55	52.4	0.27	0.77	56.2	0.51	0.61

*Among those with Type 2 Diabetes (n = 2,284). Response based on whether subjects felt very confident in managing their diabetes well.

### 4.2 Multivariable results

#### 4.2.1 T2D prevalence

In Table 2, our first model shows that Latinos had a higher unadjusted odds of having T2D if they reported low food security (Model 1: OR = 1.36; 95% CI:1.11–1.67) or very low food security (OR = 1.47; 95% CI:1.11–1.94) compared to their food-secure counterparts. The odds ratios grew after controlling for covariates (Model 2: low food insecurity: OR = 1.44; 95% CI:1.14–1.83; very low food security: OR = 1.87; 95% CI:1.33–2.61). In models stratified by nativity/duration, Latinos born in the US had a higher odds of having T2D if they reported low food security (Model 3: OR = 1.60; 95% CI:1.02–2.52) or very low food security (OR = 2.37; 95% CI:1.45–3.86) compared to food secure US Born Latinos. However, among Latinos living in the US for <10 years, there was no statistically significant difference between food security status and T2D prevalence. Lastly, among Latinos living in the US for 10 years or more, those who reported low food security (OR = 1.48; 95% CI:1.12–1.97) or very low food security (OR = 1.78; 95% CI:1.15–2.76) had a higher odds of having T2D compared to their food-secure counterparts.

**Table 2:** Odds of Self-Reported Type 2 Diabetes by Food Security Status among Latino Adults That Live Under 200% of the US Federal Poverty Line, California Health Interview Survey 2012–2017, N = 16,254.

	**Overall sample**						**Stratified by Nativity/Duration of US Residence**								
	**Model 1**			**Model 2**			**Model 3**			**Model 4**			**Model 5**		
	**Unadjusted**			**Adjusted**			**US Born, Adjusted Model**			**Immigrants < 10 Years in US, Adjusted model**			**Immigrants ≥ 10 Years in US, Adjusted model**		
	**OR**	**[95% CI]**		**OR**	**[95% CI]**		**OR**	**[95% CI]**		**OR**	**[95% CI]**		**OR**	**[95% CI]**	
**Food Security Status**															
High / Marginal Food Security^a^															
Low Food Security	1.36**	1.11	1.67	1.44**	1.14	1.83	1.60*	1.02	2.52	0.56	0.11	2.75	1.48**	1.12	1.97
Very Low Food Security	1.47**	1.11	1.94	1.87**	1.33	2.61	2.37**	1.45	3.86	1.07	0.20	5.76	1.78**	1.15	2.76
**Duration in the United States**															
Born in US^a^															
<10 Years				0.44*	0.23	0.82									
10 Years+				0.84	0.64	1.11									
**Covariates**															
Age				1.06**	1.05	1.07	1.07**	1.06	1.08	1.08**	1.05	1.12	1.05**	1.04	1.06
Gender															
Female^a^															
Male				1.27	0.92	1.75	1.29	0.86	1.93	1.12	0.32	3.91	1.28	0.83	1.98
Year															
2012^a^															
2013				1.24	0.89	1.74	2.09*	1.14	3.84	0.08**	0.02	0.38	1.13	0.76	1.68
2014				0.97	0.7	1.35	2.04*	1.05	3.95	0.50	0.09	2.93	0.77	0.52	1.13
2015				1.07	0.78	1.48	1.42	0.76	2.63	0.30	0.06	1.67	1.02	0.70	1.50
2016				1.1	0.77	1.58	0.98	0.50	1.93	0.44	0.05	4.06	1.19	0.79	1.79
2017				1.14	0.8	1.62	0.96	0.45	2.04	1.61	0.03	3.86	1.24	0.82	1.88
Educational Attainment															
Less than High School^a^															
High School Diploma				0.72*	0.56	0.94	0.69	0.41	1.16	0.30	0.05	1.78	0.76	0.57	1.02
Some College +				0.62	0.38	1.01	0.71	0.26	1.90	0.44	0.03	5.66	0.58	0.32	1.06
Income Category															
<20,000^a^															
20000–29999				1.05	0.81	1.36	0.94	0.60	1.49	0.8	0.08	7.97	1.08	0.81	1.46
30,000+				0.97	0.72	1.31	1.39	0.78	2.46	0.34	0.01	7.91	0.86	0.60	1.23
Family Type															
Single No Children^a^															
Married No Children				1.44*	1.08	1.92	1.67	0.84	3.3	0.79	0.20	3.15	1.36	1.00	1.86
Married with Children				1.03	0.78	1.38	1.43	0.86	2.37	0.30	0.06	1.6	0.91	0.62	1.33
Single with Children				0.83	0.55	1.26	1.46	0.73	2.94	0.05*	0.00	0.78	0.62+	0.37	1.05
Receiving Health Insurance				1.58**	1.20	2.07	1.27	0.63	2.56	0.92	0.20	4.33	1.72**	1.24	2.39

^a^Reference category.

Test of statistical significance=*p <.05 **p <.01.

#### 4.2.2 Perceived diabetes self-management

Table 3 provides the results for the perceived T2D self-management outcomes. In the unadjusted model, Latinos with T2D had a lower odds of reporting perceived T2D self-management if they had low food security (Model 1: OR = 0.62, 95% CI:0.42–0.92) or very low food security (OR = 0.45, 95% CI:0.28–0.73) compared to those who were food secure. In Model 2, these associations remained statistically significant after controlling for covariates. In models stratified by nativity, among Latinos born in the US, there was no association between food security status and perceived T2D self-management (Model 3). Finally, Latinos born outside the US had a lower odds of reporting that they managed their T2D well if they reported low security (Model 4: OR = 0.54; 95% CI:0.34–0.86) or very low food security (OR = 0.36; 95% CI:0.17–0.74) compared to their food-secure counterparts.

**Table 3:** Odds of Perceived Self-Management of Type II Diabetes (T2D) among Latino Adults That Live Under 200% of the US Federal Poverty Line and have T2D, California Health Interview Survey 2012–2017, N = 2,284.

	**Overall Sample**						**Stratified by Nativity**					
	**Model 1**			**Model 2**			**Model 3**			**Model 4**		
	**Unadjusted**			**Adjusted**			**US Born, Adjusted Model**			**Foreign-Born, Adjusted Model**		
	**OR**	**[95% CI]**		**OR**	**[95% CI]**		**OR**	**[95% CI]**		**OR**	**[95% CI]**	
**Food Security Status**												
High/Marginal Food Security^a^												
Low Food Security	0.62*	0.42	0.92	0.56**	0.36	0.86	0.72	0.31	1.72	0.54**	0.34	0.86
Very Low Food Security	0.45**	0.28	0.73	0.46**	0.26	0.83	0.82	0.32	2.09	0.36**	0.17	0.74
**Born in US**				0.63	0.38	1.04						
**Covariates**												
Age				1.00	0.98	1.02	0.98	0.96	1.01	1.01	0.99	1.04
Gender												
Female^a^												
Male				1.46*	1.03	2.07	0.98	0.41	2.34	1.69*	1.07	2.66
Year												
2012^b^												
2013				1.20	0.67	2.12	2.28	0.87	5.97	0.92	0.45	1.91
2014				1.05	0.59	1.89	1.15	0.43	3.07	1.08	0.52	2.24
2015				0.98	0.54	1.80	1.18	0.32	4.40	0.89	0.48	1.64
2016				1.24	0.67	2.29	1.62	0.44	5.96	1.08	0.54	2.16
2017				1.07	0.52	2.20	2.47	0.56	10.89	0.77	0.36	1.67
Educational Attainment												
Less than High School^a^												
High School Diploma				0.95	0.61	1.48	0.68	0.28	1.62	1.03	0.59	1.79
Some College +				1.31	0.52	3.28	1.09	0.20	5.96	1.09	0.40	2.98
Income Category												
<20,000^a^												
20000–29999				0.93	0.59	1.48	1.02	0.32	3.23	0.92	0.54	1.58
30,000+				0.73	0.38	1.43	1.35	0.38	4.82	0.73	0.31	1.68
Family Type												
Single No Children^a^												
Married No Children				0.94	0.59	1.50	1.49	0.50	4.45	0.78	0.41	1.47
Married with Children				0.75	0.40	1.40	0.38	0.09	1.65	0.87	0.37	2.04
Single with Children				0.48	0.22	1.04	0.46	1.79	1.79	0.58	0.20	1.64
Receiving Health Insurance				1.21	0.72	2.02	5.08	0.96	27.30	0.97	0.51	1.87

^a^Reference category. Test of statistical significance= *p <.05 **p <.01.

## 5 Discussion

We examined if food insecurity was associated with T2D prevalence and perceived T2D self-management among Latinos living in California. We also explored if these associations differed by nativity and duration of residence in the US. We found that Latinos had a higher odds of T2D if they reported low food security and very low food security, in a graded fashion, compared to Latinos who were food secure. Latinos born in the US and foreign-born Latinos living in the US for 10 years or greater exhibited a similar trend, with food insecurity associated with higher odds of T2D. However, this relationship was not present among Latinos living in the US for <10 years.

Our findings suggest that food insecurity is an important factor that influences T2D susceptibility for US-born Latinos and longer-stay Latino immigrants. Responses to food insecurity, especially pertaining to dietary intake, may differ between US-born and longer-stay Latino immigrants compared to more recently arrived immigrants. For example, like many other socio-economically disadvantaged groups in the US, food-insecure Latino immigrants may cope with their food insecurity similarly to US-born Latinos by selecting and consuming cheap, low-quality, energy-dense foods (Darmon and Drewnowski, 2008). These types of foods exacerbate weight gain, insulin resistance, and thus risk of T2D. In contrast, recent Latino immigrants experiencing food insecurity may consume staple foods that are similar to their country of origin, including corn tortillas, beans, eggs, and tomatoes (Kaiser et al., 2003). Buffering the negative effects of food insecurity. In general, independent of food insecurity, longer stay Latino immigrants have been shown to have worse dietary intake than recent immigrants (Ayala et al., 2008, Pérez-Escamilla, 2011). However, more recent evidence suggests that dietary acculturation may have a greater role in some heritage groups than others given the vast heterogeneity in culture, heritage, and diet among Latinos (Maldonado et al., 2021).

Alternatively, food insecurity may act as an independent stressor that exacerbates insulin resistance and thus T2D. Both acute (Li et al., 2013) and chronic stress have been found to be associated with glucose metabolism and insulin resistance. Recent research has found that among Latinos with T2D, inflammation and stress biomarkers mediate the association between household food insecurity and insulin resistance (Bermúdez-Millán et al., 2019). The psychological stressful state of being food insecure may increase inflammation as well as cortisol levels, resulting in these metabolic outcomes. Although recent immigrants experience higher levels of food insecurity, longer stay immigrants potentially have longer exposures to food insecurity and thus higher levels of chronic stress.

The null association between food insecurity and T2D among recent immigrants may also be attributed to immigrant health selection. That Latino immigrants have lower T2D prevalence in general than their US-born counterparts, with recent immigrants having the lowest prevalence, is often attributed to the selectivity hypothesis of immigrant health (Schneiderman et al., 2014, Cho et al., 2004). This hypothesis argues that immigrants are selected on characteristics, such as better baseline health and health-promoting behaviors, that make them more likely to migrate compared to those left behind in the country of origin. These characteristics contribute to better post-migration health outcomes compared to the US-born and explain their positive health outcomes despite experiencing risk factors such as low socioeconomic status (Riosmena et al., 2013). However, this health advantage tends to decrease with a longer duration of living in the United States (Cho et al., 2004). The findings of this paper align with this theory; such that recent immigrants in our sample were less likely to have T2D compared to their longer stay counterparts. Similar patterns have been found for obesity, with recent immigrants, displaying a null association between food insecurity and obesity, despite having the highest levels of food insecurity (Ryan-Ibarra et al., 2017). In contrast, food insecurity is associated with a higher likelihood of obesity among those born in the US and immigrants with longer duration in the US (Ryan-Ibarra et al., 2017). Additionally, independent of health selection, it’s possible that the null association between food insecurity and T2D among recent immigrants may be due to the long latency period of T2D (CDC, 2022) and poor access to healthcare among new immigrants leaving them undiagnosed (Barcellos et al., 2012). Often, there is an asymptomatic period during the early stages of T2D which can go unnoticed and undiagnosed (CDC, 2022).

In terms of perceived T2D self-management, Latinos with T2D had lower odds of managing their T2D well if they had low or very low food security, compared to their food-secure counterparts. However, after stratifying by nativity, we found this relationship only existed among foreign-born Latinos. These analyses did not examine differences among immigrants by duration of residence because of the small sample sizes for foreign-born Latinos with diabetes living in the US for <10 years. Based on the distribution of T2D in our sample, the results for perceived T2D self-management are mostly drawn from the longer stay immigrant Latinos. These findings on perceived T2D self-management run counter to our findings on the relationship between FI and T2D prevalence; US-Born Latinos were more likely to have T2D when food insecure, but did not have lower odds of good perceived T2D self-management when food insecure. One reason why we see worse perceived T2D self-management among food insecure immigrant Latinos, but not food insecure US-born Latinos, may be related to the utilization of health care services. Past research has found that immigrant Latinos are less likely to utilize health care services, especially private and primary care clinic visits (Alcalá et al., 2016). Even though we controlled for health insurance, US-born Latinos who have diabetes may have better clinical support in managing their diabetes than immigrant Latinos.

There are limitations to this study. First, this study utilizes cross-sectional data which cannot determine causality or directionality of the models. Second, all of our measures were self-reported and thus are at risk of recall and social desirability biases (California Health Interview Survey | UCLA Center for Health Policy Research, 2017). T2D was measured by a respondent reporting whether a physician told them they have T2D. By nature, this form of measurement excludes respondents who may not have access to a physician, and may thus be undiagnosed. We controlled for health insurance status in our models to possibly adjust for this. Individuals with undiagnosed T2D are less likely to have regular access to care and more likely to be low-income and represent a high proportion of the Latino population in the US (Alcalá et al., 2016). This may result in underestimating Latinos with T2D, thus making our findings conservative. Perceived T2D self-management was measured by asking respondents how confident they are in controlling and managing their T2D. It is possible that people reported being confident about their self-management even though they may have poor control. Future studies should utilize longitudinal designs and use clinical measurements to determine T2D status and T2D self-management.

## 6 Conclusions

In this study of Latinos in California with incomes <200% of the federal poverty line, we found that food security (FS) status was associated with type 2 diabetes (T2D) prevalence and perceived T2D self-management. However, these associations varied according to nativity and US duration of residence. Latinos born in the United States and longer stay immigrant Latinos (10 years or more in the US) both had a higher odds of having T2D when food insecure compared to their food-secure counterparts. We found that for perceived T2D self-management, there were no differences by food security status among Latinos born in the United States, but there were among Latino immigrants. Latino immigrants who were food insecure had a lower odds of managing their T2D well compared to their food-secure counterparts with T2D.

## 7 Implications for research and practice

This study shows that food insecurity may be a significant risk factor for type 2 diabetes among Latinos. Before intervening on prediabetes and T2D, healthcare providers should screen for food insecurity. Additionally, policy efforts and community-based interventions should focus on increasing food security among Latinos. Future research should focus on pathways between food insecurity and T2D and T2D self-management, such as dietary pathways, and how these pathways might vary among Latinos of different nativity and duration of residence in the US. This research will be useful in developing targeted interventions aimed at reducing T2D among Latinos and improving proper self-management among Latinos with T2D. Future interventions could target recently arrived food-insecure Latino immigrants to prevent the onset of T2D by reducing food insecurity. Additionally, health interventions for immigrant Latinos with T2D should address unmet material needs, such as food insecurity, in order to improve self-management of T2D.

## Author contributions

Osborn designed the research study, conducted analyses, and wrote the draft. Albrecht and Ro guided the design of the study. Ro guided the analysis and provided language help. Albrecht, Fleischer, and Ro critically revised the content of the article. All authors provided final approval before submission.

## Funding

Support was provided to the second author by National Institute of Diabetes and Digestive and Kidney Diseases, grant no. NIH/NIDDK K01DK107791.

## CRediT authorship contribution statement

**Brandon Osborn:** Conceptualization, Methodology, Software, Formal analysis, Investigation, Data curation, Writing – original draft, Writing – review & editing. **Sandra S. Albrecht:** Methodology, Validation, Writing – review & editing, Resources. **Nancy L. Fleischer:** Methodology, Writing – review & editing. **Annie Ro:** Methodology, Software, Investigation, Writing – review & editing, Supervision.

## Declaration of Competing Interest

The authors declare that they have no known competing financial interests or personal relationships that could have appeared to influence the work reported in this paper.
